# Hypertensive Heart Failure

**DOI:** 10.3390/jcm12155090

**Published:** 2023-08-02

**Authors:** Filippos Triposkiadis, Pantelis Sarafidis, Alexandros Briasoulis, Dimitrios E. Magouliotis, Thanos Athanasiou, John Skoularigis, Andrew Xanthopoulos

**Affiliations:** 1 School of Medicine, European University Cyprus, 2404 Nicosia, Cyprus; 2 Department of Nephrology, Hippokration Hospital, Aristotle University of Thessaloniki, 54124 Thessaloniki, Greece; 3 Department of Therapeutics, Heart Failure and Cardio-Oncology Clinic, National and Kapodistrian University of Athens, 11527 Athens, Greece; 4 Unit of Quality Improvement, Department of Cardiothoracic Surgery, University of Thessaly, 41110 Larissa, Greece; 5 Department of Surgery and Cancer, Imperial College London, St Mary’s Hospital, London W2 1NY, UK; 6 Department of Cardiology, University Hospital of Larissa, 41110 Larissa, Greece

**Keywords:** heart failure, hypertension, ejection fraction, autonomic imbalance, sympathetic nervous system

## Abstract

Despite overwhelming epidemiological evidence, the contribution of hypertension (HTN) to heart failure (HF) development has been undermined in current clinical practice. This is because approximately half of HF patients have been labeled as suffering from HF with preserved left ventricular (LV) ejection fraction (EF) (HFpEF), with HTN, obesity, and diabetes mellitus (DM) being considered virtually equally responsible for its development. However, this suggestion is obviously inaccurate, since HTN is by far the most frequent and devastating morbidity present in HFpEF. Further, HF development in obesity or DM is rare in the absence of HTN or coronary artery disease (CAD), whereas HTN often causes HF per se. Finally, unlike HTN, for most major comorbidities present in HFpEF, including anemia, chronic kidney disease, pulmonary disease, DM, atrial fibrillation, sleep apnea, and depression, it is unknown whether they precede HF or result from it. The purpose of this paper is to provide a contemporary overview on hypertensive HF, with a special emphasis on its inflammatory nature and association with autonomic nervous system (ANS) imbalance, since both are of pathophysiologic and therapeutic interest.

## 1. Introduction

Hypertension (HTN) is the leading cause of cardiovascular disease and premature death worldwide, which largely surpasses other important factors of mortality such as smoking and metabolic diseases [1,2,3]. HTN is the most important risk factor for heart failure (HF) development, with recent evidence indicating that HTN is present in 76% of incident HF cases [4], and the lifetime risk of HF is almost twice as high in people with HTN as in those with normal blood pressure (BP) [5,6]. From a pathophysiological standpoint, HTN causes left ventricular (LV) hypertrophy (LVH), fibrosis, and structural alterations of large and small arteries (microvascular disease) [7]. Further, several epidemiological studies have revealed the association between HTN and coronary artery disease (CAD), a major HF risk factor [7]. In this regard, in the INTERHEART study, 25% of the population-attributable risk of a myocardial infarction could be accounted for by HTN [8].

**Figure 1:**
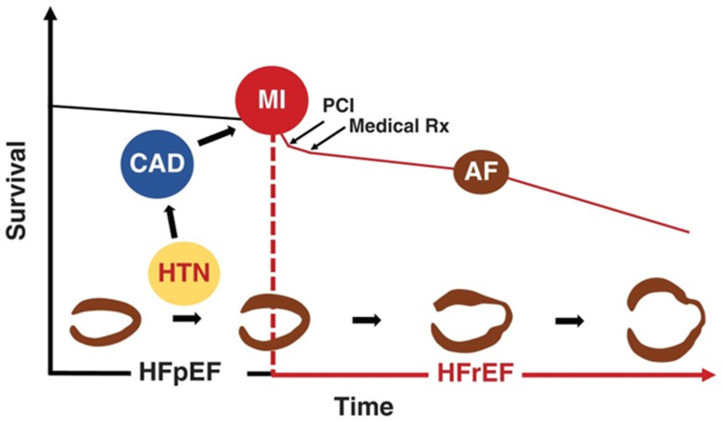
The importance of hypertension (HTN) in heart failure (HF) pathogenesis. A patient with HTN may develop concentric left ventricular (LV) hypertrophy (LVH), which is a risk factor for HF with preserved ejection fraction (HFpEF) that may progress to eccentric LVH and HF with reduced ejection fraction (HFrEF). Alternatively, as HTN is a major risk factor for coronary artery disease (CAD), the patient may suffer an acute myocardial infarction (MI). Following MI, primary coronary angioplasty (PCI) and medications (Medical Rx) attenuate myocardial damage and favorably affect prognosis. Eventually, however, eccentric LVH and HFrEF may develop, and the outcome may be further compromised by incident atrial fibrillation (AF), which frequently appears in the setting of LV dysfunction and, in turn, adversely affects LV structure, function, and outcome. With permission from Ref. [21].

Despite overwhelming epidemiological evidence, the contribution of HTN to HF development has been undermined in current clinical practice. This is due to the fact that approximately half of HF patients have been labeled as suffering from HF with preserved left ventricular (LV) ejection fraction (EF) (HFpEF), with HTN, obesity, and diabetes mellitus (DM) being considered virtually equally responsible for its development [9]. However, this is inaccurate, since HTN is by far the most frequent and devastating morbidity present in HFpEF, with its prevalence reaching 80% in the Get With the Guidelines (GWTG) initiative [10], and 90% or more in large randomized clinical trials testing the effectiveness of medical treatment in HFpEF [11,12,13,14]. Further, HF development in obesity or DM is rare in the absence of HTN or CAD [15,16,17,18], whereas HTN often causes HF per se [19]. Finally, unlike HTN, for most major comorbidities present in HFpEF including anemia, chronic kidney disease, pulmonary disease, DM, atrial fibrillation (AF), sleep apnea, and depression, it is unknown whether they precede HF or result from it [20,21], and combinations of morbidities (multimorbidity) might occur randomly because the individual component conditions are common. Due to these drawbacks, comorbidity was recently redefined as the accumulation of additional morbidities to an index morbidity (a specific morbidity under consideration) over an individual’s lifetime [22]. This approach is of practical importance because it is crucial to discerning the settled mechanisms leading to multimorbidity, as chains of disease causation might be separated in time (e.g., antecedent HTN may give rise to CAD and myocardial infarction, which in turn can later lead to HF complicated by AF) (Figure 1) [21].

The purpose of this paper is to provide a contemporary overview on hypertensive HF (HHF), defined as the presence of HF symptoms and signs in a patient with a history of HTN and elevated systolic blood pressure (BP) at presentation (≥130 mmHg) [23] in the absence of an acute coronary syndrome or a history of it, and hemodynamically significant valvular or congenital heart disease. Special emphasis will be given to the inflammatory nature of HHF and its association with autonomic nervous system (ANS) imbalance, since both are of pathophysiologic and therapeutic interest. Throughout this manuscript, the referenced HFpEF studies are those in which the results were driven by HTN.

## 2. Pathophysiology of Hypertension 

The pathophysiological mechanisms responsible for HTN are complex, and act on a genetic background. Further, the probability of developing HTN increases with aging, due to the progressive stiffening of the arterial wall caused by, among other factors, slowly developing changes in vascular collagen and increases in atherosclerosis [24].

The mosaic theory of hypertension, which has prevailed to the present day, proposes that HTN pathophysiology is multifaceted. Accordingly, HTN is caused by multiple factors, including genetics, environment, adaptive, neural, mechanical, and hormonal perturbations, which intertwine to elevate BP [25,26]. Over the years, the Mosaic paradigm has been modified, and new concepts such as oxidative stress, inflammation, sodium homeostasis, and the microbiota have arisen, giving rise to further refinements of the ,osaic theory [27]. 

One of the recently proposed pathways for BP elevation involves the gut microbiota. The microbiota and the brain (i.e., the microbiota–gut–brain axis) communicate via various routes including the immune system, the vagus, and the enteric nervous system. Many factors can influence microbiota composition in early life, including infection, type of birth delivery, antibiotics, nutritional provision, environmental stressors, and host genetic factors. On the other hand, microbial diversity decreases with aging [28].

**Figure 2:**
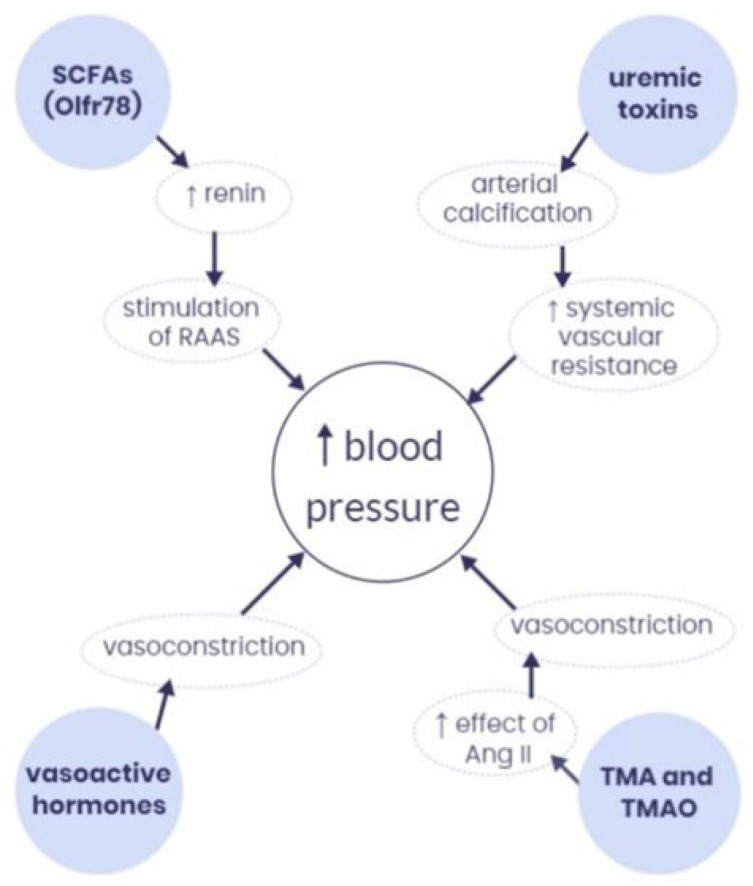
Interaction between microbiota-derived metabolites and blood pressure. SCFAs—short-chain fatty acids; RAAS—renin–angiotensin–aldosterone system; Ang II—angiotensin II; TMA—trimethylamine; TMAO—trimethylamine N-oxide, Olfr78—olfactory receptor 78, ↑—increase. With permission from Ref. [29].

There is compelling evidence that shifts in gut microbiota play a key role in BP regulation. In this regard, intestinal bacteria synthesize metabolites, the most important being short-chain fatty acids (SCFAs), vasoactive hormones, trimethylamine (TMA) and trimethylamine N-oxide (TMAO) and uremic toxins, such as indoxyl sulfate (IS) and p-cresyl sulfate (PCS) [29] (Figure 2) [29]. Microbiota derangements are causally associated with HTN [30]. Hypertensive stimuli (stress, diet, salt, maternal factors, environmental toxins, etc.) significantly impact the microbiota-gut-brain axis and influence ANS brain regions to affect sympathetic, endocrine, and immune pathways [31]. Chronic overactivation of immune cells either directly or through the gut microbiota ultimately produces chronic inflammation and HTN. T cells are central to the immune responses underlying HTN, as activated T cells infiltrate tissues and produce cytokines including interleukin 17A, which promote renal and vascular dysfunction as well as end-organ damage, thereby leading to HTN [32].

In the gut itself, hypertensive stimuli cause gut microbial dysbiosis, gut barrier weakening, and inflammation [31]. Weakened barrier function allows previously excluded gut contents (e.g., bacteria and their metabolites) to come in contact with the immune system and generate inflammation, which in turn exacerbates leakiness and inflammation and recruits pro-inflammatory bone marrow progenitor cells to the gut generating a vicious cycle. The factors crossing the weakened gut barrier and inflammatory mediators generated in the gut reach the brain via the circulation and contribute to the development of local (neuroinflammation) and systemic inflammation [33]. 

Finally, there is bidirectional interaction between the microbiota and the renin angiotensin system (RAS), a major determinant of BP levels. Gut bacteria and their metabolites modulate gastrointestinal and systemic RAS, and at the same time, changes in the intestinal habitat caused by alterations in RAS may shape microbiota metabolic activity and composition [34]. Thus, an inflammatory milieu developed by hypertensive stimuli precedes, and is causally related to HTN development regardless of the presence or absence of other morbidities, rendering the theory of multimorbidity-induced inflammation leading to HFpEF obsolete [35]. It comes, therefore, as no surprise that HFpEF in the absence of HTN is rare [36].

The effect of the aforementioned perturbations on BP levels is also affected by genetic factors. Several genes have been identified to play a role in the pathophysiology of HTN, including those involved in the renin–angiotensin–aldosterone system (RAAS), catecholamine/adrenergic system, renal kallikrein–kinin system, epithelial sodium channel, adducin, and those involving lipoprotein metabolism, hormone receptors, and growth factors [37]. Genome-wide association studies (GWAS) have exposed more than 100 variants associated with blood pressure in the general population [38], and some studies have reported genes associated with hypertensive heart disease’s complications such as cardiomyopathy and HF [39]. Lastly, post-genomic biomarkers, from the emerging fields of transcriptomics, proteomics, glycomics, and lipidomics, have provided new insights into the molecular underpinnings of hypertension [37].

## 3. From Hypertension to Hypertensive Heart Failure 

HTN is characterized by chronic LV pressure overload and increased intravascular volume, both affecting the LV structure and function [40]. Nevertheless, the conventional concept, described more than 120 years ago by William Osler, has been that HTN leads to concentric LV hypertrophy (LVH), which in turn is followed by eccentric LVH and HF [41]. In this regard, Messerli et al. proposed that HHF develops in four stages: [42] (a) stage I: isolated LV diastolic dysfunction with no LV hypertrophy; (b) stage II: LV diastolic dysfunction with concentric LVH; (c) stage III: clinical HF (dyspnea and pulmonary edema) with concentric LVH; and (d) stage IV: eccentric LVH with HF and reduced ejection fraction. 

However, doubt has been raised that the previously mentioned sequence of events is typical in hypertensives, based on the following arguments: (a) concentric LVH may not be the most frequent geometric pattern, and is less commonly seen than eccentric LVH in studies enrolling hypertensive subjects [43]; (b) the transition from concentric LVH to eccentric LVH is uncommon in the absence of CAD [44]; (c) the risk varies by LV geometric pattern, with eccentric and concentric LVH predisposing individuals to HFrEF and HFpEF, respectively [45].

However, recent studies have provided compelling evidence that cardiac remodeling is a dynamic process, with phenotypic transitions occurring frequently regardless of the presence or absence of CAD. For example, in the Swedish Heart Failure Registry (SwedeHF), the proportion of patients with HFpEF (approximately 70% were hypertensive) that worsened during follow-up (from HFpEF to HF with mid-range LVEF [HFmrEF] or HF with reduced LVEF [HFrEF]) was 31.2% in the absence of baseline CAD, 33.1% in the presence of baseline CAD, and 57.2% in the case of interim CAD [46]. Similar were the findings of another study including 1082 patients (approximately 70% were hypertensive) admitted to hospital due to decompensated HFpEF (LVEF > 50% at the first LVEF assessment at discharge). At LVEF reassessment within 6 months in the outpatient setting, 758 patients (70%) had an LVEF > 50%, 138 patients (13%) had an LVEF of 40–49% (HFmrEF), and 186 patients (17%) had an LVEF of <40% (HFrEF) [47]. In addition, antihypertensive treatment attenuates or even reverses cardiac remodeling [48,49].

**Figure 3:**
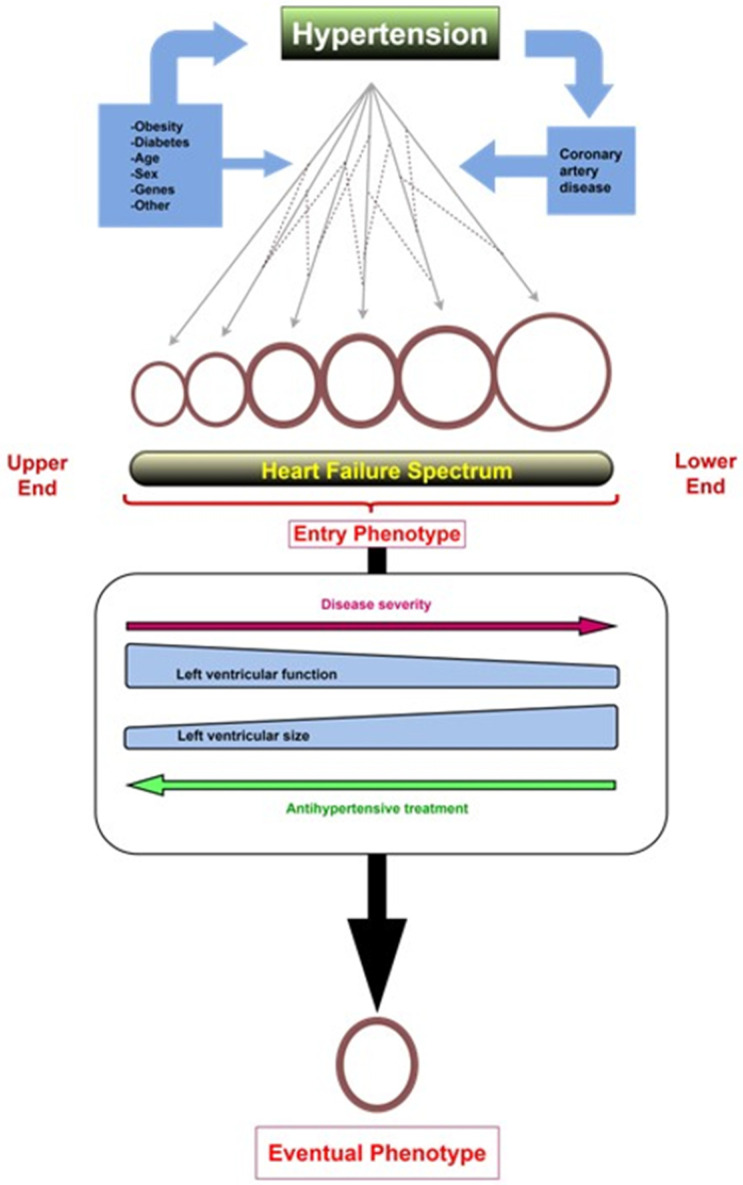
Cardiac remodeling in hypertensive heart failure (HHF). According to the spectrum theory, each HF phenotype results from a patient-specific trajectory where the heart remodels towards concentric left ventricular hypertrophy (LVH), eccentric LVH, or a combination of the two. In the case of hypertension (HTN), the port of entry in the HF spectrum (HHF entry phenotype) depends on (a) HTN severity, duration and antihypertensive treatment effectiveness; (b) the balance between LV pressure and LV volume overload; (c) the coexistence of morbidities such as obesity, diabetes mellitus, and coronary artery disease; and (d) disease modifiers (age, sex, genes, other). The eventual HHF phenotype results from transitions across the HF spectrum, whose direction predominantly depends on disease severity and antihypertensive treatment, which shift towards the lower end of upper end of the HF spectrum, respectively.

We have developed and promoted the concept of the HF spectrum across phenotypes, according to which each HF phenotype results from a patient-specific trajectory wherein the heart remodels towards concentric LVH, eccentric LVH, or a combination of both [50,51,52]. We believe that this concept fully explains the diversity of cardiac remodeling observed in HHF (Figure 3). The port of entry in the HF spectrum (HHF entry phenotype) depends on (a) HTN severity, duration and antihypertensive treatment effectiveness; (b) the balance between LV pressure and LV volume overload; (c) the coexistence of morbidities such as obesity, DM, and CAD that preexist and/or modify LVH development; and (d) disease modifiers (age, sex, genes, other). The eventual HHF phenotype results from transitions across the HF spectrum, whose direction depends on disease severity and antihypertensive treatment, which shift towards the lower end of upper end of the HF spectrum. 

## 4. Cardiac Autonomic Imbalance 

HTN evolves over the lifespan, from predominant sympathetic nervous system (SNS)-driven HTN with elevated mean BP in early and mid-life to a late-life phenotype of increasing systolic and falling diastolic BP, associated with increased arterial stiffness and aortic pulsatility [53]. However, growing evidence indicates that the SNS is also capable of modulating arterial stiffness independently of prevailing hemodynamics and vasomotor tone [54,55]. The contribution of arterial stiffness to HTN and HF development is high in the elderly and patients with CKD [53,56]. 

The autonomic dysregulation observed in HTN escalates in HHF. SNS overactivity has long been appreciated as a compensatory mechanism initially supporting the failing heart, which, however, in the long term, triggers a sequence of unfavorable remodeling processes, causing HF progression and the occurrence of major cardiovascular events [57,58]. 

The adverse cardiac effects of SNS overactivity in HF have been predominantly studied in dilated LV with eccentric LVH, in which it manifests as an increase in norepinephrine spillover from the cardiac sympathetic endings, leading to chronic β-adrenergic receptor (AR) hyperstimulation and maladaptive GRK2 upregulation (GPCR kinase 2) [59], thereby promoting β-AR down-regulation, cardiac hypertrophy, and myocyte apoptosis. Further, GRK2 recruits β-arrestin, which then competes with G-proteins for interaction with the β-AR and limits their activation [60]. The chronic SNS overactivity In HF with eccentric LVH has been attributed to several neurogenic disturbances acting in concert [61]. 

SNS overactivity is also present in HF with nondilated hearts and lack of eccentric LVH. In this regard, earlier studies suggested that the SNS overdrive which is present in essential HTN is augmented in HF without eccentric LVH [62,63]. These findings have also been confirmed in recent HFpEF studies, in which most patients were hypertensive. Kaye et al. evaluated 14 healthy volunteers and 20 HFpEF patients (65% hypertensive), and found systemic sympathoexcitation in HFpEF patients, as indicated by the increased plasma arterial norepinephrine concentration and plasma levels of dihydroxyphenylglycol (a major intraneuronal metabolite of recaptured norepinephrine) compared with normal controls [64]. Seo et al., using iodine-123-labeled metaiodobenzylguanidine (123I-MIBG) single-photon emission computed tomography (SPECT) imaging in 148 patients admitted for acute decompensated nonischemic HFpEF (91% hypertensive) [65], observed that during a mean follow-up period of 2.4 ± 1.6 years, those with a high total defect score (TDS) levels had a significantly greater risk of cardiac events than those with middle or low TDS levels.

**Figure 4:**
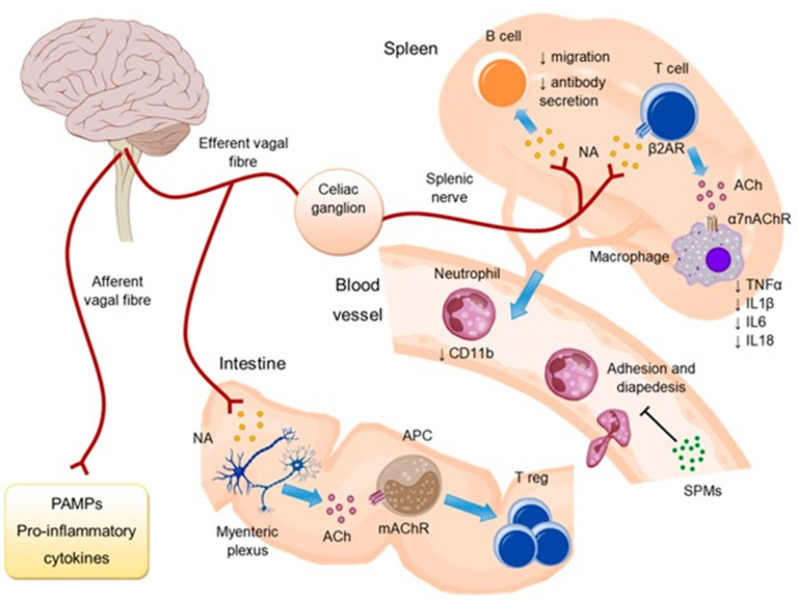
The parasympathetic nervous system-mediated anti-inflammatory reflex. Vagal afferents respond to inflammation mediators and send signals to the brainstem, where a signal is produced and transmitted by vagal efferents to the splenic nerve, causing noradrenaline (NA) release in the spleen. CD4+ T lymphocytes, which express the β2- adrenaline receptor (β2AR), uptake NA and release acetylcholine (ACh). Acetylcholine inhibits the synthesis of proinflammatory cytokines in the macrophages that express the α7nAChR receptor (α7 nicotinic acetylcholine receptors). Further, the anti-inflammatory reflex diminishes CD11b expression on neutrophils, augments the release of pro-resolving mediators (SPMs), and reduces antibody secretion and migration of B lymphocytes. In the intestine, ACh stimulates antigen-presenting cells (APCs) through muscarinic receptors (mAChR), contributing to the maintenance of regulatory T cells (Treg). With permission from Ref. [67].

In contrast to SNS overactivity, there is attenuation of the parasympathetic nervous system (PNS) activity and its physiological effects in HF [66], including the PNS-mediated anti-inflammatory reflex (Figure 4) [67]. The vagus nerve also plays a crucial role in this reflex, providing the afferent and efferent pathways which underly the communication between the brain and peripheral organs, including the heart [67,68]. Vagal sensory afferents are activated by proinflammatory cytokines in peripheral tissues and convey the signal to the brain. Subsequently this signal causes the release of acetylcholine from vagal efferents into the reticuloendothelial system, which inhibits proinflammatory cytokine synthesis and release. Thus, vagal withdrawal contributes to the creation of the inflammatory milieu in HF [69]. 

## 5. Treatment of Hypertensive Heart Failure 

In patients with HF, treatment of HTN has been unanimously recommended, but BP targets have been inappropriately defined [70]. However, as RAAS and SNS overactivity play key roles in HTN and HF as well in the development of major complications such as kidney dysfunction, their inhibition is of outmost importance for the management of HHF [71]. Further, another class of agents, the sodium glucose cotransporter 2 inhibitors (SGLT-2i), which have proved tremendously effective in HF management, have a complex pleiotropic mechanism of action, including a reduction of neurohormonal overactivity [72].

### 5.1. Blood Pressure Targets

In the major outcome trials of hypertension, comparison of BP reduction with antihypertensive medications against placebo or no treatment, incident HF was the outcome that showed the largest intergroup risk reductions [73,74,75]. Effective HTN treatment in HF should target not only average BP, but the time in therapeutic range pressure range (TTR) as well. 

#### 5.1.1. Target Average Blood Pressure

It has been suggested that there is a J-shaped curve describing an inverse relationship between BP and cardiovascular complications, and that this association is more pronounced in patients with preexisting CAD, HTN, or LVH [76]. However, several lines of evidence dispute the presence of such a curve. 

The target BP which should be pursued with medical treatment was evaluated in the pivotal SPRINT (Systolic Blood Pressure Intervention Trial), which demonstrated that among patients at high risk for cardiovascular events in the absence of DM, targeting a systolic BP < 120 mmHg (intensive treatment group; mean systolic BP at one year 121.4 mmHg), as compared with < 140 mmHg (standard treatment group; mean systolic BP at one year 136.2 mmHg), decreases the incidence of the primary outcome (myocardial infarction, other acute coronary syndromes, stroke, HF, or death from cardiovascular causes) [77]. Nevertheless, analyses comparing the effects of intensive and standard BP treatment in the ACCORD (Action to Control Cardiovascular Risk in Type 2 Diabetes) trial showed that the diabetic patients who received standard glycemic therapy and intensive BP control had benefits similar to those seen in SPRINT [78,79]. 

The results of SPRINT have been confirmed in several subsequent investigations. which demonstrated a significant and direct dose–response between systolic BP levels and ischemic heart disease risk across all systolic BP exposure values (100–200 mmHg), without evidence for a J-shaped curve [80,81].

According to the 2018 ESC/ESH guidelines, in hypertensive patients with HF, BP-lowering treatment should be considered if an individual’s BP is ≥140/90 mmHg, and systolic BP should be lowered to a range of 120–130 mmHg [7].

#### 5.1.2. Time in Therapeutic Range

Although BP is a continuous and dynamic variable, a single or average BP value has been used for BP monitoring in clinical practice and HTN studies. To overcome this limitation, it has been recommended that doctors should pursue every point of BP monitoring instead of measuring a single value of BP [82]. In this regard, the term TTR was introduced, which expresses the percentage of BP measurements recorded within a certain window (e.g., TTR for BP window 120–140 mmHg) and reflects, therefore, the prevailing BP during the follow-up period and the magnitude of BP variability [83] (Table 1). The importance of TTR in HTN management was documented in a study including 371,996 hypertensive patients, in which the mortality rate increased from the most consistently controlled quartile (>75% in TTR) towards the less consistently controlled quartiles [83]. Likewise, a secondary analysis of the TOPCAT (Treatment of Preserved Cardiac Function Heart Failure With an Aldosterone Antagonist) trial, in which the TTR was calculated with the target range of systolic BP defined as 110 to 130 mmHg [84], a greater time in the systolic BP target range was associated with a decreased risk of cardiovascular outcomes and mortality events beyond BP level, especially among younger patients. Finally, in a recent post hoc analysis of HHF patients both from TOPCAT and BEST (Beta-Blocker Evaluation of Survival Trial) showed a linear relationship between TTR and the primary outcome (cardiovascular death or HF hospitalization), and similar patterns were observed in the individual trials [85]. Moreover, sensitivity analyses redefined target range as 110 to 130 mmHg for systolic BP or 70 to 80 mmHg for diastolic BP. 

Based on the above, we believe that a systolic BP target range of 110–130 mmHg with a TTR > 75% should be pursued in all HHF patients regardless of LVEF, provided it is well tolerated. These BP targets may also be applied in elderly HHF patients, as well as in those with coexistent DM [86] or kidney disease [87].

### 5.2. Medications 

The drugs used in the management of HF, except for diuretics, have both cardioprotective and blood pressure-lowering properties (Table 2). 

#### 5.2.1. RAAS Inhibitors (RAASi)

RAASi, that is, ACEi, ARBs, sacubtril/valsartan (ARNI), and MRAs, effectively reduce BP in hypertensive patients [88]. The aforementioned agents have also proved effective in HF regardless of the LVEF [89,90,91]. Interestingly, in a post hoc analysis of PARAGON-HF (Prospective Comparison of ARNI With ARB Global Outcomes in HF With Preserved Ejection Fraction), ARNI was found to be useful in treating apparent resistant hypertension in patients with HFpEF, even in those who continued to have an elevated BP, despite treatment with at least four antihypertensive drug classes, including an MRA [92].

Steroidal MRAs, such as spironolactone and eplerenone, have proved effective in patients with HF, resistant HTN, or CKD [93]. However, the associated risk of hyperkalemia and hormonal side effects limit their broad implementation, especially in patients with type 2 DM and moderate-to-advanced chronic CKD [94]. 

**Figure 5:**
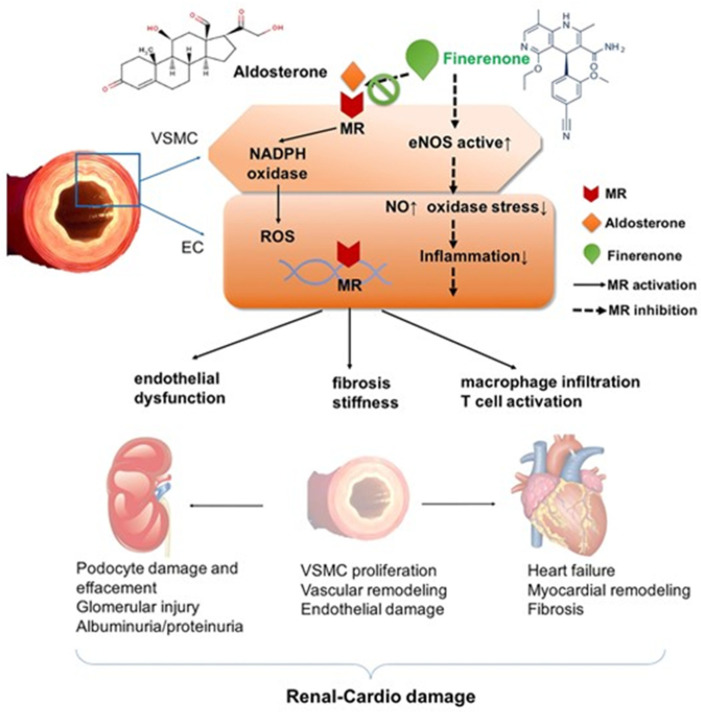
Renal and cardiac damage induced by mineralocorticoid receptor (MR) overactivity. MR overactivity stimulates NADPH oxidase and enhances ROS accumulation in VSMCs and ECs, increasing oxidative stress. MR agonists (e.g., aldosterone) promote endothelial dysfunction, macrophage infiltration and T cell activation, as well as cytokine collection, leading to VSMC fibrosis and stiffening. MR overactivity adversely affects kidney function, by aggravating podocyte damage and effacement, injuring the glomerulus injury, and promoting VSMC proliferation and endothelial damage, which lead to vascular remodeling. Regarding the heart, MR overactivity pro-motes myocardial remodeling and fibrosis, thereby exacerbating heart failure. In contrast, the nonsteroidal MR antagonist finerenone blocks the binding of aldosterone and MR, and attenuates the cardiac derangements induced by MR overactivity. MR, mineralocorticoid receptor. EC, endothelial cells. VSMC, vascular smooth muscle cells. ROS, reactive oxygen species. NADPH, nicotinamide adenine dinucleotide phosphate. With permission from Ref. [98].

In addition to the beneficial effects on the heart, ACEi/ARB, ARNI may slow progression to kidney failure, and thus they are appropriate for initial therapy for managing HHF, especially, and coexistent DM [95]. Finerenone is a novel, nonsteroidal MRA which significantly reduces the risk of hard cardiovascular and kidney failure outcomes with a minimal risk of hyperkalemia in hypertensive patients with type two DM and a broad range of CKD, as demonstrated in the FIDELIO-DKD (Finerenone in Reducing Kidney Failure and Disease Progression in Diabetic Kidney Disease; 97% hypertensive patients) trial [96] and the FIGARO-DKD (Finerenone in Reducing Cardiovascular Mortality and Morbidity in Diabetic Kidney Disease; 96% hypertensive patients) trial (Figure 5) [97,98]. 

Finally, as many patients with HHF and DM may manifest a resistant form of HTN, the use of ARNI instead of an ACEi/ARB together with an MRA may be considered. If BP remains elevated, antihypertensive drugs with complementary mechanisms of action and lack or minimal negative inotropy (e.g., diuretics or dihydropyridine calcium channel blockers) should be progressively added to lower BP [99]. Subgroup analyses of these trials also provided preliminary evidence that the efficacy and safety profile of finerenone was similar, irrespective of background therapy with other medications such as SGLT-2i (see below). In the ARTS-DN (Mineralocorticoid Receptor Antagonist Tolerability, Study-Diabetic Nephropathy) trial (823 patients [approximately 95% hypertensive] with type 2 DM and CKD, with an urine albumin-to-creatinine ratio 30 mg/g and an estimated glomerular filtration rate of, 30–90 mL/min per 1.73 m^2^ had a reduced daytime and night-time SBP according to 24 h ambulatory blood pressure monitoring [100].

#### 5.2.2. Sodium Glucose Cotransporter 2 Inhibitors (SGLT-2i)

**Figure 6:**
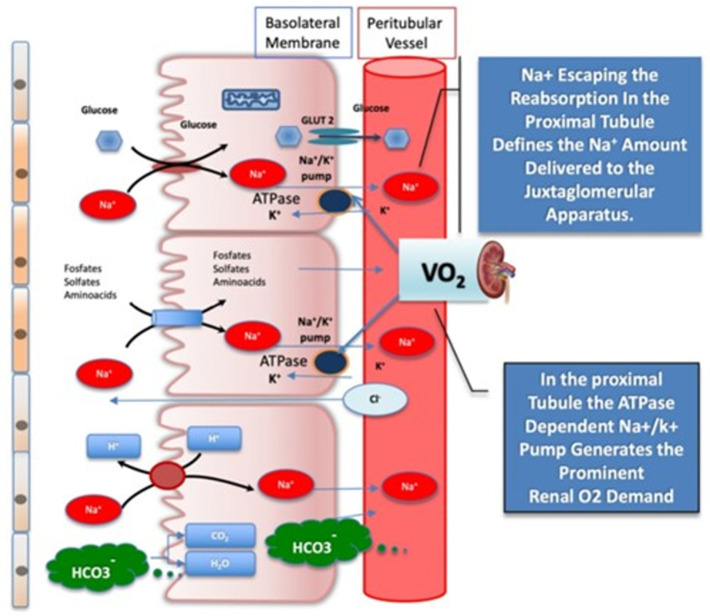
Filtrates reabsorbed by the sodium–glucose co-transporter (SGLT) 2, which operates at the glomerular proximal tubule. SGLT2, bicarbonate reabsorption, and the Na^+^/K^+^ ATPase, which provides energy to drive both, are depicted. The active reuptake of bicarbonate and of other solutes, coupled with Na^+^, drives the high tissue O_2_ consumption in the kidney. Na^+^ escaping the reabsorption at the proximal section tubule is sensed by the juxtaglomerular apparatus and sets the afferent arteriole tone. GLUT2 = glucose transporter 2; SGLT2 = sodium–glucose co-transporter 2. With permission from Ref. [102].

The SGLT-2i block the sodium–glucose cotransporter 2, which is located on the apical membrane of the proximal convoluted tubules, and therefore target glucose reabsorption (Figure 6) [101,102]. The SGLT-2i also reduce blood pressure, but their underlying mechanism(s) is(are) incompletely understood. Potential explanations include (a) an osmotic effect of glucose, allowing more sodium and water to remain within the tubules, causing natriuresis [103]; (b) excess glucose and sodium excretion, leading to RAAS Modification [104]; and (c) attenuation of SNS overactivity [105,106].

Recent clinical trials have reported that SGLT-2i have a beneficial effect on BP, which is not just an acute effect of treatment initiation, but has a long-term impact on both systolic and diastolic BP [107]. Further, SGLT-2i significantly reduces the rate of cardiovascular events (including HF) and prevents the progression of renal dysfunction and CKD development in patients with or without DM already receiving optimal medical therapy [108]. However, the reduction in BP in these trials was “modest”, and not of a magnitude to fully account for the approximately 30–40% reduction in HF, end-stage kidney disease, or renal or cardiovascular mortality [109], providing further evidence in support of the pleiotropic effects of SGLT-2i [110]. 

In addition to HTN, SGLT-2i have proved tremendously effective in reducing morbidity and mortality in HF patients irrespective of LVEF and diabetic status [111,112]. Regarding the SGLT-2i effects in HF, a recent meta-analysis of 16 RCTs reported that SGLT-2i were associated with a statistically significant reduction in systolic BP of 1.68 mmHg, no significant change in diastolic BP, a 1.36 kg decrease in body weight, a 0.16% decrease in glycated hemoglobin level, a 1.36% increase in hematocrit, and no change in heart rate [113]. The mechanisms underlying the beneficial effects of SGLT-2i in HF have not been delineated. According to an interesting hypothesis, the SGLT-2i rebalance the reabsorption of Na^+^ coupled with glucose and restore renal O2 demand, diminishing neuroendocrine activation [102]. Alternatively, SGLT-2i, by alleviating inflammation and oxidative stress, down-regulate hepcidin, upregulate transferrin receptor protein 1, and reduce ferritin, the net result being an increase the levels of cytosolic Fe^2+^ available to mitochondria, thus enabling the synthesis of heme in erythroid precursors and ATP in cardiomyocytes [114]. 

#### 5.2.3. Beta Adrenergic Receptor Blockers (BBs)

BBs lower BP as effectively as other major antihypertensive drugs and have solid documentation in preventing cardiovascular complications [115]. BBs can be broadly classified into (a) nonselective β-blockers (e.g., propranolol) with similar β1 and β2 activity (none of the β-blockers belonging to this class are indicated for HF); (b) β1-selective blockers with a higher affinity for β1-adrenoreceptors (metoprolol, bisoprolol, and nebivolol), preferred in patients with chronic obstructive pulmonary disease or mild asthma (nebivolol also facilitates nitric oxide release, causes vasodilation, and is preferred in patients with HTN); and (c) β-blockers with additional α-1-adrenoreceptor antagonism and consequent peripheral, vasodilation (carvedilol), preferred in patients with HTN or documented higher peripheral vascular resistance.

Although BBs have proved to be lifesaving in HHF with eccentric LVH [116], they have never been appropriately tested in HF with concentric LVH [117], presumably due to the misbelief that the latter is not associated with SNS overactivity, which dominated for decades [118]. Moreover, it has been argued that pharmacological heart rate lowering in HHF patients with concentric LVH may have adverse effects on hemodynamics, exercise capacity, and outcomes [119]. Accordingly, it is our contention that vasodilating BBs (nebivolol and carvedilol) should be used for protection from excessive ventricular rates in atrial fibrillation, prophylaxis of tachyarrhythmias, symptomatic treatment of effort angina, and resistant HTN. Nevertheless, these complications often occur in HHF patients [36,120] and it comes, therefore, as no surprise that in recent large HFpEF trials, approximately 80% of study participants were treated with BBs [12,13,14]. 

#### 5.2.4. Diuretics

Loop diuretics, which inhibit the Na^+^/K^+^/2Cl^−^ symporter at the ascending limp loop of Henle, have the most potent diuretic effect, promote excretion of sodium and chloride (and potassium, albeit to a lesser extent than thiazides), and form the backbone of diuretic therapy in congestive HF [121]. Adequate dosing with sufficient plasma levels is pivotal as renal perfusion is often reduced in HF, resulting in diminished secretion of loop diuretics. 

Thiazide diuretics are most commonly used to treat HTN, although they can be adjuncts in HF management on top of loop diuretics in patients with diuretic resistance [122]. They inhibit the Na^+^/Cl^−^ symporter in the distal convoluted tubule, leading to decreased sodium and water reabsorption. They may prescribed for patients with HHF following decongestion instead of loop diuretics due to (a) their efficacy and low side effect profile; (b) their synergistic effect when combined with other antihypertensive agents; and (c) their counteraction of salt and fluid retention caused by other antihypertensive agents [123].

#### 5.2.5. Implementation of Medical Treatment

The optimal time frame from initiation of antihypertensive therapy to attaining the levels of BP control that influence cardiovascular outcomes is not well defined. Overall, a series of landmark trials in hypertensive patients and additional cardiovascular risk factors collectively support the prompt achievement of BP control, ideally within 1–3 months [124].

**Figure 7:**
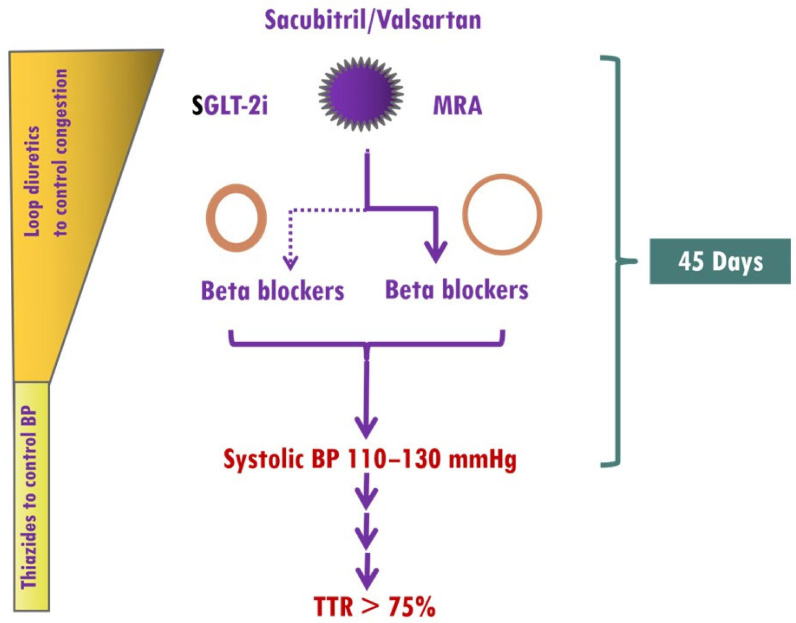
Implementation of medical treatment in hypertensive heart failure (HHF). The presence of elevated BP in HHF as well as the renoprotective effects of finerenone, which rarely causes early hyperkalemia, allows ultra-fast up-titration of HF medications. Treatment should start with the simultaneous use of sacubitril/valsartan, sodium glucose cotransporter 2 inhibitors (SGLT-2i), and mineralocorticoid receptor antagonists (MRAs, preferably finerenone). In HFF patients with eccentric left ventricular hypertrophy (LVH), BBs (preferably vasodilatory) should be started from the beginning, whereas in HHF patients with concentric LVH, BBs should be considered in those with atrial fibrillation, coronary artery disease, or resistant hypertension. A target systolic blood pressure of 110–130 mmHg should be achieved within 45 days, and thereafter, systolic blood pressure should be within the target range.

The main obstacles to up-titration of medications in HF patients include a low BP, impaired renal function, electrolyte imbalance, and drug intolerance, which are frequently encountered during optimization of HF medications. However, the presence of elevated BP in HHF as well as the renoprotective effects of finerenone, which rarely causes early hyperkalemia [125], allows ultra-fast up-titration of HF medications. Thus, treatment should start with the simultaneous use of sacubitril/valsartan, SGLT-2i and finerenone. In HFF patients with eccentric LVH, BBs should be started from the beginning, whereas in HHF patients with concentric LVH, BBs should be used in selected patients, as previously mentioned (Figure 7). 

A majo© iss©e impeding ©he implementation of optimal medical treatment of HTN and HF is poor patient adherence to the prescribed drugs [126]. In this regard, treatment with fixed-dose combination pills may improve patient’s adherence and cardiovascular outcomes. An observational retrospective study of three Italian Local Health Units demonstrated that only half of patients prescribed atorvastatin–perindopril–amlodipine as a free combination were adherent to the medical therapy, whereas it was estimated that 69,542 hypertensive patients could be eligible for a fixed dose of atorvastatin–perindopril–amlodipine during 2014 [127]. An open-label multicenter study including 356 patients with HTN and/or CAD from 17 centers in Poland between January 2015 and August 2016, showed that a fixed-dose combination of bisoprolol and aspirin was associated with excellent or good (≥76%) adherence in 98.3% and 98.0% of patients based on pill counts and patients ‘diaries, respectively. A significant decrease was also observed in mean systolic BP, mean diastolic BP, and heart rate over the 3-month period (all *p* < 0.001) [128]. Many new drug combinations have been approved over the last few years, allowing the development of new single-pill formulations. The use of single-pill drugs seems to be optimal in the treatment of patients with HHF and concomitant diseases such as hyperlipidemia, AF, and DM.

### 5.3. Exercise

**Figure 8:**
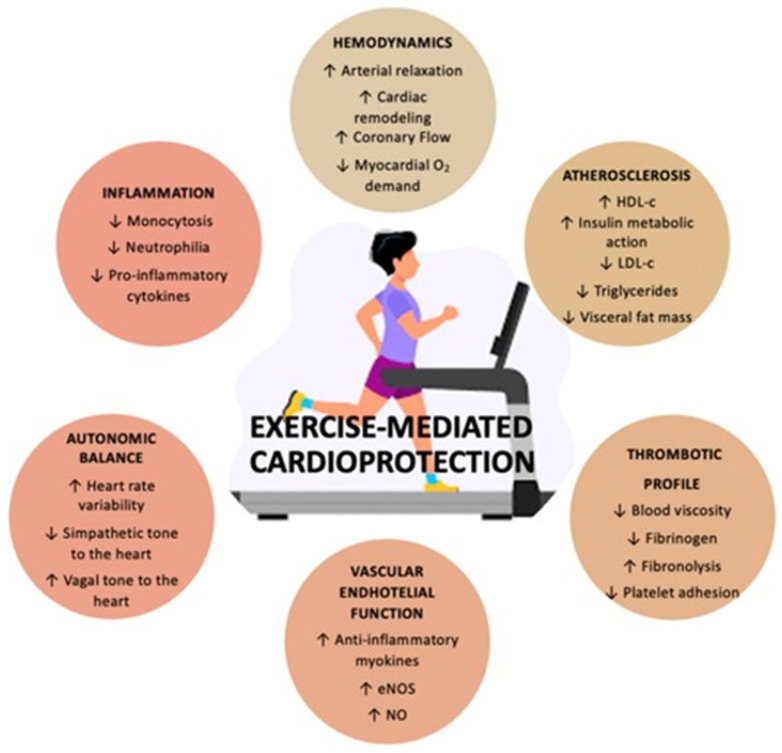
The protective effects of exercise on the cardiovascular system. Abbreviations: eNOS: endothelial nitric oxide synthase; NO: nitric oxide; HDL-c: high-density lipoprotein cholesterol; LDL-c: low-density lipoprotein cholesterol. With permission from Ref. [130].

Exercise training is internationally recommended for patients with HF, regardless of LVEF [129]. Optimal exercise prescription enhances exercise capacity, improves quality of life, and reduces hospitalizations and mortality in HF patients. Specifically, physical activity promotes cardiovascular health by (Figure 8) [130,131,132,133] (a) attenuating the negative effect of many established risk factors for cardiovascular disease (cholesterol, insulin sensitivity, body mass index, cholesterol, BP, sleep apnea); (b) directly influencing the cardiovascular system (increasing nitric oxide bioavailability, enhancing endothelial function, favoring arterial wall remodeling consisting of increased diameter and dilation capacity, decreasing coronary and wall thickness, developing coronary collateral vessels, and stabilizing coronary atheromatic plaque); (c) restoring the ANS balance and protecting against fetal arrhythmias; and (d) exhibiting antithrombotic and anti-inflammatory effects. Importantly, exercise interventions based on aerobic, combined, or isometric exercise are probably the most important currently available means to decrease arterial stiffness in adults with HTN [134].

### 5.4. Emerging Treatments

As the gut microbiota and their metabolites may contribute to HTN development, they represent a reasonable treatment target [135]. Since the gut microbiota is a complex and highly interactive system for the treatment of HTN, a “one-size fits all” approach using a metabolite or bacterial strain is unlikely to restore the perturbed metabolic activity associated with intestinal dysbiosis in HTN. Instead, probiotic supplementation using diverse bacterial strains producing or reducing the production of certain metabolites to elicit an antihypertensive effect is a potential treatment strategy for HTN management. There is evidence to suggest that intervention with probiotics could lower BP, modify the intestinal flora to increase the abundance of beneficial bacteria, and regulate intestinal microbial metabolites such as trimethylamine oxide, short-chain fatty acids, and polyphenols [136]. A recent umbrella meta-analysis which assessed the pooled effect size of 14 meta-analyses with 15,494 participants indicated significant decreases in both systolic and diastolic BP following probiotics supplementation. Greater effects on SBP were revealed in trials with a mean age of >50 years and the duration of intervention ≤10 Weeks [137]. DBP was also more reduced in studies with a dosage of ≥1010 colony-forming units (CFUs), and SBP was decreased in patients with HTN or DM, when analyzing mean difference. Gut microbiota science has far-reaching clinical implications for the diagnosis, prognosis, and monitoring of cardiovascular diseases in general, and HHF in particular. Dietary supplementation remains the safest probable method to improve the gut microbiota and its associated detrimental cardiovascular effects.

As increased SNS and decreased PNS activities are associated with HTN and HF, the neuromodulatory therapies of renal denervation (RDN) and vagus nerve stimulation (VNS) have received recent attention [138]. Catheter-based RDN can be used as an adjunct treatment option in uncontrolled resistant HTN despite best efforts toward lifestyle and pharmacological interventions, as well as in patients intolerant to antihypertensive medications in the long term [139]. A shared decision making process is a key feature, and preferably includes a patient who is well informed on the benefits and limitations of the procedure. Further studies are necessary to answer questions related to the impact of BP lowering with RDN on clinical outcomes and potential clinical indications beyond RDN. As the vagus nerve plays a critical role in the inflammatory reflex [140], VNS could attenuate the proinflammatory state known to be a key pathological mechanism in HTN and HHF, and attenuate cardiac remodeling. It is of interest that VNS can be performed in a less invasive manner via transcutaneous stimulation of the auricular branch of the vagus nerve, using the tragus of the ear as an anatomical landmark. In a recent study, HFpEF patients (96% hypertensive) were randomized to either active (tragus) or sham (earlobe) low-level transcutaneous vagus nerve stimulation (20 Hz, 1 mA below discomfort threshold), for 1 h daily for 3 months [141]. After three months, diastolic function improved and inflammatory markers decreased. 

## 6. Perspective 

There is no doubt that in most patients with HF, many morbidities coexist (multimorbidity). This is partly because HF patients are elderly, and the prevalence of multimorbidity dramatically increases with age (especially after the age of 70). In this regard, as the prevalence of several of these morbidities is high in the community, their coexistence in elderly HF patients may be due to chance. Alternatively, many cardiac and non-cardiac morbidities (e.g., anemia, DM, sleep apnea, kidney dysfunction and failure, atrial fibrillation, etc.) may be present because of cardiac dysfunction. Therefore, it is highly probable that most of the morbidities present in HF did not have the opportunity to contribute to the inflammatory environment that led to HF; instead, they resulted from it. In contrast, HTN always precedes not only the development of HF but CAD as well, which together with HTN are by far the most common and important risk factors for incident HF. Further, as convincingly demonstrated in recent studies, HTN may develop in an inflammatory environment created in the absence of other morbidities, especially in the young. LVEF-based classification of HF phenotypes has served well over the years. However, the time has come to realize that its continuation may lead to erroneous misconceptions, one of them being the mislabeling of HHF patients as suffering from HFpEF; this may undermine the importance of HTN, both in HF’s development and as treatment target. As exercise training is one of the few interventions that may reduce arterial stiffness, it should be an integral part of HHF management. 

## Figures and Tables

**Table 1:** Example illustrating the importance of time in therapeutic range (TTR) in hypertension management. Although the average systolic blood pressure (BP) achieved with treatment is similar among the three patients, the TTR (target range for systolic BP: 110–130 mmHg) significantly differs.

Systolic BP	Patient A	Patient B	Patient C
**Measurement 1**	**135**	**125**	**115**
**Measurement 2**	**125**	**125**	**120**
**Measurement 3**	**135**	**120**	**125**
**Measurement 4**	**140**	**120**	**120**
**Measurement 5**	**100**	**140**	**125**
**Measurement 6**	**100**	**100**	**135**
**Measurement 7**	**125**	**125**	**115**
**Measurement 8**	**135**	**115**	**125**
**Measurement 9**	**135**	**140**	**135**
**Measurement 10**	**105**	**100**	**120**
**Average BP (mmHg)**	**123.5**	**121**	**123. 5**
**Time to target (%)**	**30% (3/10)**	**60% (6/10)**	**80% (8/10)**

**Table 2:** Therapeutic targets and medications in the management of hypertensive heart failure.

	Congestion	Blood Pressure Lowering and Cardioprotection
	**Loop diuretics** **(furosemide, torasemide,** **bumetanide)**	**ACEi/ARB/Sacubitril-valsartan** **Steroidal MRAs (spironolactone, eplerenone)** **B-blockers**
**Blood Pressure Targets** ** *Systolic BP* ** ** *110–130 mmHg* ** ** *Time in therapeutic range > 75 %* **	**Comments:** **1. Additional use of thiazide diuretics or carbonic anhydrase inhibitors in cases with diuretic resistance** **2. Thiazide diuretics may be considered in decongested patients instead of loop diuretics for blood pressure control.**	**Comments:** **1. Besides effectively lowering BP, all the aforementioned classes of antihypertensives are cardioprotective. ** **2. Vasodilating beta-blockers with a favorable metabolic profile (e.g, carvedilol, nebivolol) may be preferable. ** **3. Beta-blockers are first-line agents in eccentric hypertrophy. ** **4. B-blockers should be used in selected patients with concentric hypertrophy (e.g., atrial fibrillation, angina, resistant hypertension).** **5. The additional use of dihydropyridine calcium channel blockers should be considered when BP cannot be otherwise controlled.**

BP, blood pressure; ACEi, angiotensin-converting enzyme inhibitor; ARB, angiotensin receptor blocker; MRA, mineralocorticoid receptor antagonist.

## Data Availability

Not applicable.
